# 

**Published:** 1908-10

**Authors:** 


Texas Health Officers Association. Official Report.

VOL. XXIV. OCTOBER, 1908. No. 4.

LIBRARY
Indexed
S. G. O.

### TEXAS MEDICAL JOURNAL

"RED BACK"

PUBLISHED AT AUSTIN, TEXAS, BY

F. E. DANIEL, M. D., - - Proprietor

PRINTED BY THE VON BOECKMANN-JONES CO.

Subscription, $1.00 a Year, in Advance. - - - Single Copies, 10 Cents

Entered at the Postoffice at Austin, Texas, as Second-class Mail Matter

